# Intensity Modulated Radiotherapy (IMRT) and Fractionated Stereotactic Radiotherapy (FSRT) for children with head-and-neck-rhabdomyosarcoma

**DOI:** 10.1186/1471-2407-7-177

**Published:** 2007-09-13

**Authors:** Stephanie E Combs, Wolfgang Behnisch, Andreas E Kulozik, Peter E Huber, Jürgen Debus, Daniela Schulz-Ertner

**Affiliations:** 1 University of Heidelberg, Department of Radiation Oncology, Im Neuenheimer Feld 400, 69120 Heidelberg, Germany; 2 University of Heidelberg, Department of Pediatric Hematology and Oncology, Im Neuenheimer Feld 153, 69120 Heidelberg Germany; 3 Cancer Research Center (dkfz), Department of Radiation Oncology, Im Neuenheimer Feld 280, 69120 Heidelberg, Germany

## Abstract

**Background:**

The present study evaluates the outcome of 19 children with rhabdomyosarcoma of the head-and-neck region treated with Intensity Modulated Radiotherapy (IMRT) or Fractionated Stereotactic Radiotherapy (FSRT) between August 1995 and November 2005.

**Methods:**

We treated 19 children with head-and-neck rhabdomyosarcoma with FSRT (n = 14) or IMRT (n = 5) as a part of multimodal therapy. Median age at the time of radiation therapy was 5 years (range 2–15 years). All children received systemic chemotherapy according to the German Soft Tissue Sarcoma Study protocols.

Median size of treatment volume for RT was 93,4 ml. We applied a median total dose of 45 Gy (range 32 Gy – 54 Gy) using a median fractionation of 5 × 1,8 Gy/week (range 1,6 Gy – 1,8 Gy).

The median time interval between primary diagnosis and radiation therapy was 5 months (range 3–9 months).

**Results:**

After RT, the 3- and 5-year survival rate was 94%. The 3- and 5-year actuarial local control rate after RT was 89%.

The actuarial freedom of distant metastases rate at 3- and 5-years was 89% for all patients.

Radiotherapy was well tolerated in all children and could be completed without interruptions > 4 days. No toxicities >CTC grade 2 were observed. The median follow-up time after RT was 17 months.

**Conclusion:**

IMRT and FSRT lead to excellent outcome in children with head-and-neck RMS with a low incidence of treatment-related side effects.

## Background

Rhabdomyosarcoma (RMS) is the most common soft tissue sarcoma entity in children [1]. The most common sites of RMS in children are the head and neck region (35%), the genitourinary tract (35%) and the extremities (17%) [2]. The orbit is the primary site in about 10% of these tumors, the most common localization in the head and neck area is parameningeal, including the nasopharynx, the paranasal sinuses, the middle ear and mastoid and the infratemporal fossa/pterygopalatine space [2-8]. Most children are younger than 10 years of age (72%) [9].

Modern therapy protocols comprise of surgical resection, chemotherapy and radiotherapy (RT). However, RT in children is commonly applied cautiously with respect to early and late side effects [10-14].

With modern RT techniques such as Intensity Modulated Radiotherapy (IMRT) and Fractionated Stereotactic Radiotherapy (FSRT), the treatment of complex shaped target volumes in close vicinity to sensitive risk structures is possible [15-17]. The present study evaluates the efficacy and toxicity FSRT and IMRT in children with head and neck RMS.

## Methods

From August 1995 to November 2005, we treated 19 children with RMS of the head-and-neck region with FSRT (n = 14) and IMRT (n = 5) at our institution.

The data presented in this analysis were acquired retrospectively and anonymously; therefore, no official approval by the local ethics committee was necessary.

### Patients' characteristics

The distribution of the tumor sites is shown in table 1. The median age of the children was 5 years at the time of RT (range 2–15 years). The male to female ratio was 1,4 : 1.

**Table 1:** Patients' characteristics of 19 children with primary head-and-neck rhabdomyosarcoma treated with FSRT or IMRT

Site	n (%)
Orbit (only)	5 (26)
Parameningeal	12 (63)
Orbit	3
Middle ear/mastoid	3
Nasopharynx/nasal cavity	6
Paranasal sinus	3
Pterygopalatine/parapharyngeal and infratempotal fossa	4
Other head and neck	2 (11)
Buccal space	1
Lip and cheek	1
Histology	
embryonal	12 (63)
alveolar	3 (16)
botryoid	4 (21)
IRS-Group	
III	17(89)
IV	2 (11)
**CWS Study Protocol**	
CWS 91	1 (5)
CWS 96	7 (37)
CWS 2002	11 (58)

Before radiation therapy, all patients had undergone at least one surgical procedure. At primary diagnosis, 3 patients had a subtotal tumor resection, and in 16 patients only a biopsy was performed.

Patients were classified according to the surgical-pathologic grouping system used in the Intergroup Rhabdomyosarcoma Study Group (IRSG) trials [18,19]. The patients' characteristics can be found in table 1.

All patients included into this study received systemic chemotherapy according to the German Soft Tissue Sarcoma Study protocols CWS 91, CWS 96 and CWS 2002 [20,21]. The aggressive interdisciplinary treatment concepts are performed in close collaboration between the radiation oncologist and the pediatric oncologist. All patients were registered at the CWS study center.

The median time interval between primary diagnosis and radiation therapy was 5 months (range 3–9 months).

### Treatment planning for FSRT and IMRT

Patients were immobilized using an individual mask fixation system made of Scotch-Cast™; for treatment planning contrast-enhanced MRI- and CT-scans were acquired with the mask attached to a non-invasive stereotactic localization frame with an overall geometrical uncertainty of 1–2 mm [22].

For FSRT, we used the three-dimensional treatment planning system Voxelplan [23]. Three to five isocentric non-coplanar irregularly shaped fields formed by a midsize multileaf collimator were applied.

**Figure 1:**
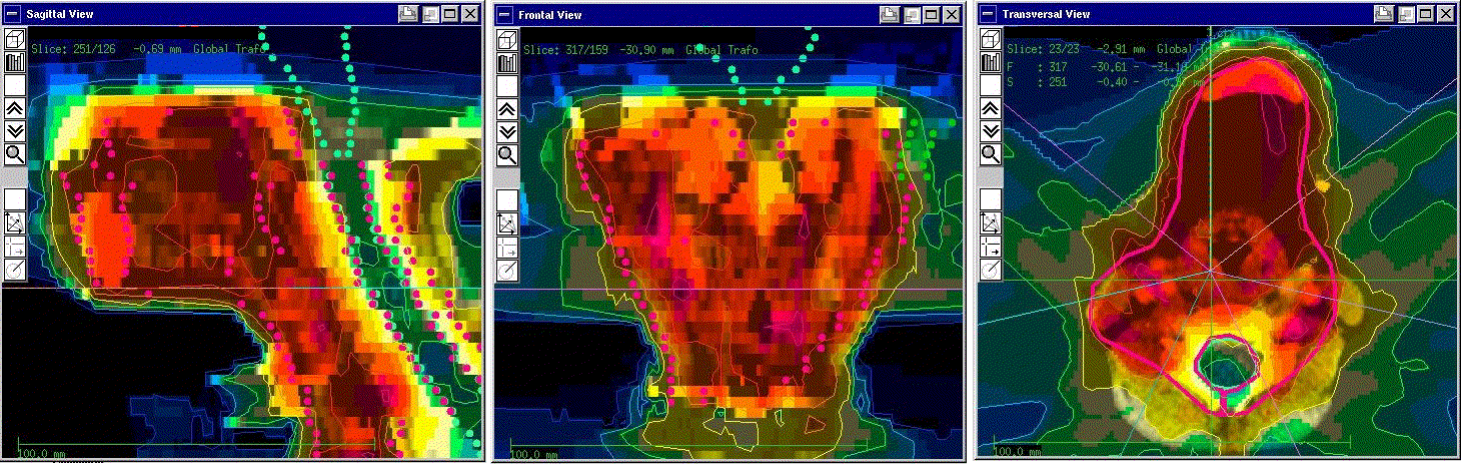
Typical 3D treatment plan of Intensity modulated Radiotherapy (IMRT) for a child with head-and-neck RMS. Thick red line = target volume; orange area 90–100% of the total dose; yellow area 70–90% of the total dose; green area 50–70% of the total dose; blue areas 10–50% of the total dose.

For IMRT, the inverse treatment-planning program Konrad (MRC Systems GmbH, Heidelberg, Germany) was used [24,25]. An integrated multi-leaf collimator allowed for IMRT using the "step-and-shoot" technique described previously [26]. Fig. 1 shows an IMRT treatment plan for a child with head and neck RMS.

Median size of the planning target volume (PTV) for radiation was 93,4 ml (range 20,8 – 354,8 ml), consisting of the area of contrast uptake in T1-weighted sequences and the enhancing lesion and edema visible in T2-weighted MRI scans prior to chemotherapy including a 20 mm safety margin. It was our policy to define the PTV according to the common guidelines outlined in the treatment protocols of the GPOH. Gross tumor volume (GTV) and clinical target volume (CTV) were not defined separately for planning purposes.

We applied a median total dose of 45 Gy (range 32 Gy – 54 Gy) using a median fractionation of 5 × 1,8 Gy/week (range 1,6 Gy – 1,8 Gy).

In 4 patients, the total dose was applied in a hyperfractionated regimen using single fraction doses of 1,6 Gy.

Deep sedation or general anaesthesia was required in 5 out of 19 children.

### Follow-up

Patients were seen for follow-up 6 weeks after radiation, thereafter in 3 to 12 months intervals, including contrast-enhanced MRI-scans, anamnesis and clinical examination. The clinical oncological follow-up was performed by the attending pediatric oncologist. Median follow-up time was 17 months (range 3 months 12 years).

### Statistics

Overall survival was calculated from primary diagnosis, survival after RT was calculated from initiation of RT. Progression-free survival after RT was calculated from the first day of RT until tumor progression (local or distant) or death (by any cause), whichever happened first, using the Kaplan Meier method [27]. All statistical analyses were performed using the Statistica 6.1 Software (StatSoft, Tulsa, OK, USA).

## Results

### Survival

**Figure 2:**
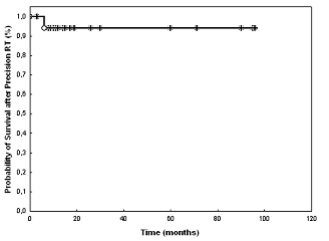
Overall survival in 19 children with head-and-neck RMS treated with FSRT or IMRT.

Overall survival rate after primary diagnosis of the RMS was 94% at 3 and 5 years, respectively (Fig. 2). One patient died of tumor progression.

### Local control

**Figure 3:**
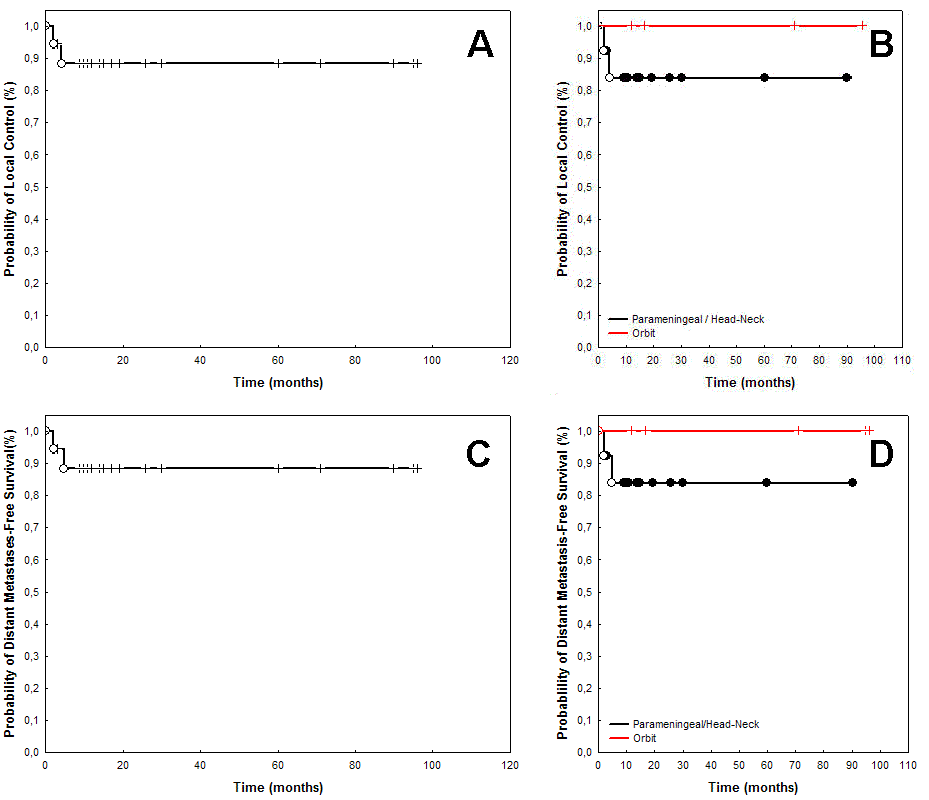
Local Control rates were 89% after 3- and 5-years (A). For RMS of the orbit only, local control was 100% after 5 years, and 81% for parameningeal RMS and other RMS of the head and neck (B). The rate of distant metastases-free survival was 89% at 5 years (C). Distant-metastases free survival rates were 100% for orbital RMS, and 81% for parameningeal and other head-and-neck RMS (D).

The 3- and 5 year actuarial local control rate after RT was 89% (Fig. 3A). We observed 2 local recurrences.

With respect to tumor localization, the 3- and 5-year local control rates were 100% for patients with RMS of the orbit and 91% for patients with parameningeal tumors. One patient with parameningeal RMS developed local progression within the prior RT field of the tumor after 4 months. The patient had received a total RT dose of 54 Gy, RT was initiated 5 months after primary diagnosis after inclusion into the CWS-91 protocol. The tumor had shown no response to the CWS chemotherapy (ifosfamide, vincristine, actinomycin-D), and the chemotherapy regimen was switched to carboplatin/etoposide, which showed also no response. Thereafter, RT was initated.

One patient with RMS (ISG Group 4) of the mylohyoid muscle developed local failure 2 months after completion of RT simultaneously with distant bone metastases and progression of the lung metastases present at primary diagnosis. This patient was 1 year old at primary diagnosis, was included into the CWS 2002 protocol and showed no response to chemotherapy prior to RT, which was initiated 4 months after diagnosis; a total RT dose of 54 Gy was applied. At primary diagnosis, lung metastases were already present.

The rate of local control in children with parameningeal and head- and-neck tumors was 81% at 3- and 5-years as compared to 100% in RMS involving the orbit only (Fig. 3B).

### Distant metastases-free survival

The actuarial freedom of distant metastases rate at 3- and 5-years was 89% for all patients at 3 and 5 years (Fig. 3C). For patients with RMS of the orbit only, the actuarial distant progression-free rate was 100% at 5 year. One patient with parameningeal RMS developed distant metastases in both lungs 6 months after completion of RT and 1 month after local tumor progression. The actuarial 3- and 5-year distant disease-free survival rate was 91%, respectively in children with parameningeal RMS. As noted above, one girl diagnosed with ISRG 4 RMS of the mylohyoid muscle developed local and distant progression simultaneously 2 months after completion of RT. The rate of distant-metastases-free survival in children with parameningeal and head-and -neck RMS was 81% at 5-years as compared to 100% in children with involvement of the orbit only (Fig. 3D).

### Toxicity

Radiotherapy was well tolerated in all children and could be completed without interruptions > 4 days.

### Acute radiation induced toxicity

Minor acute radiation-induced side effects occurring within 3 months from RT included skin erythema in 8 patients (42%), alopecia in 4 patients (21%), conjunctivitis in 3 patients (16%), mucositis in 8 patients (42%) and nausea/vomiting in 2 patients (11%); no toxicities >CTC grade 2 developed.

### Long-term radiation induced toxicity

To date, the long-term toxicity, developing per definition <6 months after RT, developed in 9 out of 19 patients (47%). We observed three patients with reduced lacrimation and one patient with chronic swelling of the left eye lid with slight erythema CTC grade 1. One patient developed trismus. However, the poor cooperation of the patient in training mouth opening after RT could have contributed to this outcome. In one patient growth hormone deficiency developed with subsequent microsomia requiring growth hormone replacement. This patient had been treated with the initial CWS-96 protocol followed by treatment according to the CWS-protocol for tumor recurrence. Radiotherapy had been applied during the initial protocol with 32 Gy, followed by 8 Gy brachytherapy and 50,4 Gy FSRT for tumor recurrence. In one patient with orbital RMS visual deficiency present prior to RT declined leading to amaurosis. Optic pathway structures received a dose of 34 Gy in 1,8 Gy fractions. Discrete erythema and swelling of the eye lid was observed in the same patient with reduced lacrimation. One patient presented with reduced mouth-opening, and facial asymmetry developed in one patient only (5%). One patient with RMS of the pterygoid fossa developed asymmetric growth of the face with growth reduction on the left side. Until now, no secondary malignancies developed.

## Discussion

Treatment guidelines of RMS include surgical resection, systemic chemotherapy using combinations of actinomycin-D, vincristine and cyclophosphamide as well as RT. Commonly, children with RMS are treated within interdisciplinary study protocols: the IRSG (North America), SIOP (Europe), CWS (Germany) and ICS protocols (Italy) [28]. Radiation therapy is an essential part in all treatment protocols for all but completely resected low risk tumors [29,30]; as a result of these multimodality approaches, the prognosis for children with RMS has improved, with long-term survival rates of 70–80%.

While improvement of outcome with respect to local and distant failures, radiation-induced side effects remained a major problem [31].

IMRT and FSRT enable the delivery of high doses to a defined target volume while sparing surrounding organs at risk; especially for complex shaped tumors of the head-and-neck, RT doses required can be applied while adhering to the tolerance doses of critical normal tissue structures. FSRT has been used safely and effectively for other childhood tumors [32,33]. IMRT helps spare normal tissues from high-dose areas, but delivers low doses to larger volumes of normal tissue discussed critically with respect to the risk of secondary malignancies. We reserved IMRT for cases in which FSRT did not yield acceptable dose distributions taking into account commonly accepted physical parameters like 90% target coverage and maximum/minimum doses to the adjacent organs at risk.

FSRT and IMRT were chosen in order to reduce dose to normal tissue structures as a general philosophy, but target volumes were not altered with different treatment techniques. Great effort was made to choose beam angles in order to reduce irradiation of bony structures not included into the target volumes in growing children. In patients where bony structures had to be included, e.g. cervical spine, symmetrical irradiation was ensured in order to avoid asymmetric bone growth whenever possible without compromises in target coverage and without exceeding the tolerance dose of normal tissue structures.

During follow-up, only one patient with RMS of the pterygoid fossa developed asymmetric growth of the face with growth reduction on the left side. It must be noted that observation time after RT is relatively short to fully determine long-term side effects such as cosmetic deformity. Therefore, longer follow-up time is needed to make final statements.

With the steep dose-fall off generated by modern high-precision RT, some concern has been raised as to whether the risk of tumor recurrences at the field border might be increased. The results obtained in the present analysis compare favourably to the results published by the large pediatric hematology study groups and reports on single-institution studies [1,34-38]. Using FSRT and IMRT, the rate of field-border recurrences seems not to be increased outside the PTV as long as the PTV itself is not reduced.

A study published by Wolden et al. reported on 28 patients with head-and-neck RMS [39]. Patients with alveolar histologic features showed significantly worse outcomes as compared to embryonal or undifferentiated tumors. However, in our study, histology as well as other established prognostic factors showed no statistically significant influence on the outcome, most probably due to the small patient number and the small number of events. Furthermore, these factors have already been addressed by the CWS trials which follow a risk-adapted combination of CHT and RT.

Proton RT is another treatment modality enabling the application of high doses while sparing surrounding normal tissue. Preliminary results of proton RT in children with orbital RMS were published by Yock et al. [40]. At the MGH in Boston, 7 children with orbital RMS were treated with proton irradiation in combination with standard chemotherapy. Treatment plan intercomparisons of proton plans and conventional 3-D plans showed that with proton RT excellent tumor coverage can be obtained while reducing the radiation dose to the adjacent normal tissues.

## Conclusion

FSRT and IMRT offer excellent results for the treatment of pediatric patients with RMS of the head-and-neck region. The incidence of acute and long-term side-effects is very low. Since only few centers can offer the treatment of pediatric patients with protons to date, FSRT and IMRT can be considered the best photon therapy modalities for a number of pediatric patients with head-and-neck RMS [40].

## Competing interests

The author(s) declare that they have no competing interests.

## Authors' contributions

SEC, WB, AEK, PEH, JD and DSE treated the patients and/or performed radiotherapy treatment planning. SEC and DSE collected the clinical data useful for the analysis; SEC and DSE performed the analysis and wrote the manuscript. WB, AEK, PEH and JD revised the article critically for important intellectual content. All authors read and approved the final manuscript.

## Pre-publication history

The pre-publication history for this paper can be accessed here:


